# Symptom Control Effectiveness of Superior Laryngeal Nerve Block in Patients with Chronic Cough: A Systematic Review and Meta-Analysis

**DOI:** 10.3390/medicina62081583

**Published:** 2026-08-18

**Authors:** Do Hyun Kim, Hye Bin Kim, David W. Jang, Se Hwan Hwang

**Affiliations:** 1 Department of Otolaryngology-Head and Neck Surgery, Seoul St. Mary’s Hospital, College of Medicine, The Catholic University of Korea, Seoul 06591, Republic of Korea; 2 Department of Head and Neck Surgery & Communication Sciences, Duke University School of Medicine, Durham, NC 27707, USA; 3 Otolaryngology-Head and Neck Surgery, Bucheon St. Mary’s Hospital, College of Medicine, The Catholic University of Korea, Seoul 06591, Republic of Korea

**Keywords:** chronic cough, neurogenic cough, laryngeal hypersensitivity, superior laryngeal nerve blocks, meta-analysis

## Abstract

*Background and Objectives:* To evaluate the efficacy of superior laryngeal nerve block (SLB) in the management of chronic cough associated with refractory laryngeal hypersensitivity. *Materials and Methods:* Studies assessing Cough Severity Index (CSI) changes before and after SLB or comparing SLB with sham interventions were included. Pooled analyses evaluated CSI reduction, subjective improvement, and adverse events. *Results:* Twelve studies involving 759 patients were analyzed. SLB significantly reduced CSI, and the improvement was maintained within the available follow-up period, with no difference between short-term (<100 days) and long-term (>100 days) follow-up (*p* = 0.5145). Most protocols used initial unilateral SLB with contralateral injection for non-responders, resulting in more than two blocks per patient on average. Subjective improvement occurred in 74%, with ≥50% CSI reduction in 55% and satisfactory response after a single block in 17%. No serious treatment-related adverse events were reported, although the available evidence was insufficient to exclude rare or delayed complications. *Conclusions:* SLB was associated with improvement in refractory chronic cough in predominantly uncontrolled observational studies. Although one small placebo-controlled trial suggested benefit over saline injection, the current evidence remains insufficient to establish definitive efficacy, and adequately powered placebo-controlled randomized controlled trials are needed to confirm its efficacy and durability.

## 1. Introduction

Chronic cough, defined as cough lasting longer than 8 weeks, affects approximately 4–10% of the general population [1]. Its etiology is multifactorial and may include respiratory infections, smoking, occupational exposures, allergic diseases, rhinosinusitis, bronchitis, asthma, eosinophilic airway inflammation, gastroesophageal or laryngopharyngeal reflux, and the use of angiotensin-converting enzyme inhibitors. Approximately 46% of patients referred to specialty clinics, including otolaryngology services, are evaluated for chronic cough [2]. After appropriate evaluation and treatment of common causes of chronic cough, including asthma, gastroesophageal reflux disease, upper airway cough syndrome, and medication-related cough, 5–10% of patients continue to experience persistent symptoms [3,4]. Depending on the diagnostic framework used, these patients have been described as having refractory chronic cough, unexplained chronic cough, chronic neurogenic cough, or laryngeal hypersensitivity cough. Although these terms are not entirely synonymous, they represent overlapping clinical populations characterized by cough hypersensitivity after exclusion of alternative causes. In recent years, increasing numbers of patients with these conditions have been referred to otolaryngology clinics, emphasizing the pivotal role of otolaryngologists in optimizing management strategies [5].

Diagnosis is largely one of exclusion, necessitating comprehensive evaluation of pulmonary function, reflux disease, allergic disorders, and medication effects [6]. A subset of patients present with a non-productive cough triggered by minimal stimuli, such as talking, laughing, positional changes, neck manipulation, eating, or exposure to air conditioning or wind [7]. Despite normal diagnostic findings for asthma, reflux, or allergies, most patients are subjected to prolonged courses of antibiotics, corticosteroids, antitussives, antihistamines, and proton pump inhibitors, often with minimal or no benefit [8,9]. This condition imposes a considerable burden, characterized by persistent symptoms, repeated diagnostic and therapeutic interventions, and diverse physical and psychosocial consequences—including urinary incontinence, cough-related syncope, and dysphonia [10,11,12,13]. Furthermore, it profoundly impairs quality of life, disrupting sleep, eating, daily functioning, and social interactions.

Superior laryngeal nerve block (SLB) using local anesthetics and corticosteroids has recently emerged as a promising therapeutic option for neurogenic cough. This minimally invasive outpatient procedure is rapid, cost-effective, well tolerated, and associated with a low risk of complications [14]. SLB may be used as an alternative or as an adjunct to behavioral and pharmacological therapies [15]. Our systematic review and meta-analysis evaluated and synthesized the existing evidence on the clinical efficacy of SLB in the management of chronic refractory cough.

## 2. Materials and Methods

This systematic review and meta-analysis was conducted in accordance with the Preferred Reporting Items for Systematic Reviews and Meta-Analyses (PRISMA) 2020 Statement. Our Institutional Review Board (Institutional Review Board of the Catholic University of Korea) approval was waived for this study, as it was a systematic review and meta-analysis conducted solely on previously published data.

### 2.1. Search Strategy and Study Selection

A comprehensive literature search was conducted in May 2026 across PubMed, Scopus, Embase, Web of Science, the Cochrane Library, and Google Scholar. Search terms were structured according to the following criteria: Patients—chronic cough, neurogenic cough, and laryngeal hypersensitivity; Intervention—SLB; Comparison—sham intervention or pre–post comparison; Outcomes—cough severity, response rate (proportion of patients reporting symptom reduction or resolution); and incidence of adverse effects (including globus sensation, injection-site pain or swelling, mild laryngospasm, throat or laryngeal paresthesia, or cutaneous purpura) (Table S1).

Two authors (DHK and HBK) independently screened the titles and abstracts of retrieved studies. When eligibility could not be determined from the title or abstract, the full text was independently assessed by a third author (DWJ). Eligible studies included prospective or retrospective investigations enrolling patients aged ≥18 years with chronic cough who underwent SLB and had validated symptom indices assessed before and after the procedure, or reported response rates or adverse effect rates.

Studies were excluded if they investigated other treatment modalities, such as respiratory retraining therapy, neuromodulating medications, or botulinum toxin injections, were duplicate publications, lacked quantitative outcome data or provided insufficient results for calculation, or used cryoablation devices that are no longer available.

Because the terminology varied across studies, we included studies describing chronic neurogenic cough, refractory chronic cough, unexplained chronic cough, sensory neuropathic cough, or laryngeal hypersensitivity cough, as defined by the original investigators, provided that patients underwent appropriate evaluation to exclude alternative causes of chronic cough before SNB. In most studies, chronic neurogenic cough was diagnosed as a diagnosis of exclusion following appropriate evaluation and management of other common causes of chronic cough. Although diagnostic protocols varied across studies, the evaluation generally included pulmonary assessment, chest imaging, assessment for gastroesophageal reflux disease, medication review (including angiotensin-converting enzyme inhibitor use), and additional allergy or speech-language pathology assessment when clinically indicated.

**Figure 1:**
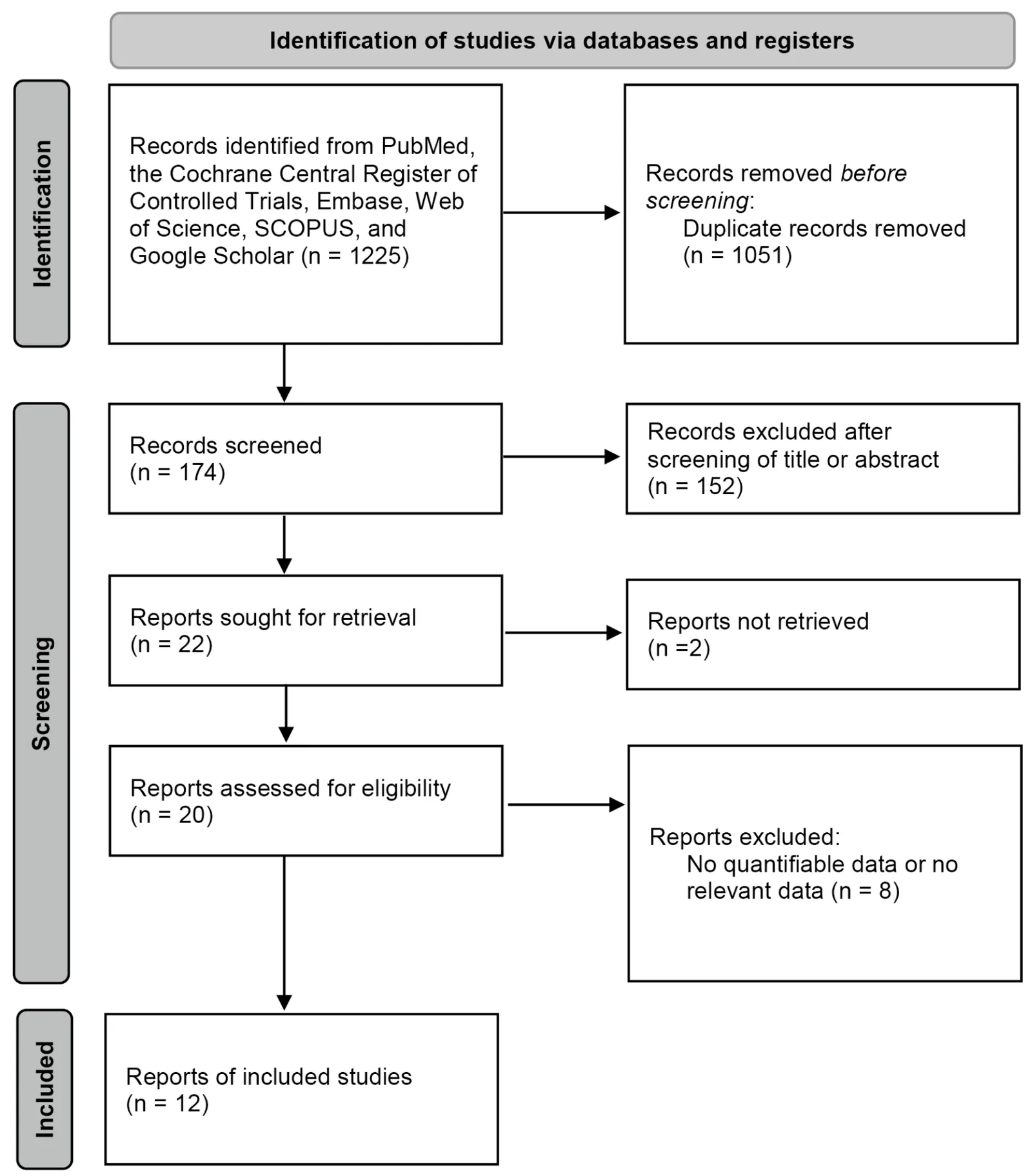
Flow diagram of the literature search and study selection.

In total, 12 studies met the inclusion criteria and were included in this systematic review and meta-analysis. The search and selection process is presented in Figure 1.

### 2.2. Data Extraction and Risk of Bias Assessment

Two authors (DHK and SHH) independently extracted data from the included studies using a standardized data collection form consistent with PRISMA 2020 guidance [16]. Potential overlap of patient populations across publications was assessed by comparing study institutions, author groups, recruitment periods, study design, and reported cohort characteristics. When overlapping cohorts were identified, only non-overlapping patient data were included in the quantitative synthesis. The Cough Severity Index (CSI) was assessed prior to SLB, with outcomes evaluated at short-term (<100 days) and long-term (>100 days) follow-up [14,15,17,18,19,20,21,22,23,24,25]. The review protocol was prospectively registered with the Open Science Framework on 21 August 2025, before study screening and data extraction commenced. The complete study protocol and dated amendment history are publicly available at https://osf.io/tq7yx/ (accessed on 21 August 2025). Comparative analyses were performed between treatment and sham groups, as well as within the treatment group before and after SLB. Detailed procedural characteristics of the SNB protocols, including injectate composition, injection laterality, injection strategy, number of procedures, interval between repeated injections, procedural guidance, and injection site, were also extracted in a standardized manner and are summarized in Table S2. In addition, whenever reported, information regarding the diagnostic work-up and treatments received before SNB, including pulmonary evaluation, gastroesophageal reflux assessment, upper airway/allergy evaluation, medication review, speech-language pathology, neuromodulator use, previous reflux therapy, and other conventional treatments, was extracted and is summarized in Table S3.

The primary outcome measures were the CSI score and the response rate, defined as the proportion of patients reporting subjective improvement in cough severity. The CSI is a validated instrument for quantifying cough-related quality of life, comprising 10 items rated on a 5-point Likert scale (0–4), with a total score ranging from 0 to 40; higher scores indicate greater symptom severity. Although widely used, a minimal clinically important difference has not been established for the CSI [19]. Subjective improvement (responder status) was determined by asking patients a binary yes/no question regarding perceived improvement in cough following SLN block.

Secondary outcomes included: (1) the proportion of patients demonstrating ≥50% quantitative improvement in cough symptoms, determined either from published patient-reported responder data or by published categorical classifications based on pre–post CSI changes reported in the original studies; (2) the proportion of patients reporting satisfaction or no further need for treatment after a single SLB; and (3) the incidence of adverse events associated with SLB. For responder analyses (≥50% improvement), only published categorical responder data were pooled. No responder rates were reconstructed from continuous outcome data or estimated using statistical assumptions. In addition, Tipton et al. [25] evaluated the efficacy of SLB compared with placebo using the Leicester Cough Questionnaire (LCQ) and a visual analog scale for cough severity, where 0 indicated complete cough resolution and 10 corresponded to baseline severity. The LCQ is a validated 19-item instrument for chronic cough-related quality of life, assessing physical, psychological, and social domains.

The methodological quality of non-randomized cohort studies was assessed using the Newcastle–Ottawa Scale (NOS) for cohort studies, which evaluates three domains: Selection (maximum 4 stars), Comparability (maximum 2 stars), and Outcome (maximum 3 stars), yielding a maximum score of 9 stars. All 11 non-randomized studies included in this review were cohort designs; accordingly, only the cohort-study version of the NOS, and none of its case–control-specific domains, was applied. Studies scoring 7–9, 5–6, and <5 stars were considered to be of high, moderate, and low methodological quality, respectively. The risk of bias in randomized controlled trials was evaluated using the Cochrane Risk of Bias tool.

### 2.3. Certainty of Evidence Assessment

The certainty of evidence for the primary efficacy and safety outcomes was assessed using the Grading of Recommendations Assessment, Development and Evaluation (GRADE) framework. The assessment considered five domains: risk of bias, inconsistency, indirectness, imprecision, and publication bias. Because most pooled estimates were derived from observational studies, the initial certainty of evidence was rated as low and was downgraded further when serious concerns were identified in one or more domains. The overall certainty of evidence was classified as high, moderate, low, or very low. Publication bias was formally assessed only for outcomes reported in at least 10 studies; for outcomes reported in fewer than 10 studies, this domain was considered not assessable.

### 2.4. Statistical Analysis

Statistical analyses were performed using R statistical software, version 4.3.0 (R Foundation for Statistical Computing, Vienna, Austria). Effect sizes were expressed as mean differences (MDs), defined as the difference in mean values either between pre- and post-treatment assessments or between treatment and control groups. MDs were calculated only when all included studies reported identical outcome measures in the same units (e.g., CSI scores). Given the expected clinical and methodological heterogeneity among the included studies, all meta-analyses were performed using a random-effects model. Between-study variance (τ^2^) was estimated using the restricted maximum-likelihood (REML) method. For uncontrolled pre–post studies, treatment effects were calculated as the mean change in CSI from baseline to follow-up, with positive values indicating symptom improvement. When the standard deviation (SD) of the change score was not reported, it was estimated from the baseline and follow-up SDs using the standard Cochrane formula:

$$SD_{\mathrm{change}}=\sqrt{SD_{\mathrm{pre}}^{2}+SD_{\mathrm{post}}^{2}-2rSD_{\mathrm{pre}}SD_{\mathrm{post}}}$$

*where*  r  *denotes the within-participant correlation coefficient.*

Because within-participant correlations were not reported in the primary studies, a correlation coefficient (r) of 0.50 was assumed to estimate the SD of the change score. Standard errors were calculated using the number of participants with paired baseline and follow-up measurements. Statistical heterogeneity was assessed using Cochran’s Q test and quantified using the I^2^ statistic. Confidence intervals for τ^2^ and τ were calculated using the Q-profile method. Publication bias was evaluated by visual inspection of funnel plots and Egger’s regression test, with the Duval and Tweedie trim-and-fill method applied when asymmetry was detected.

## 3. Results

In total, 12 studies, including 759 patients, met the inclusion criteria [14,15,17,18,19,20,21,22,23,24,25,26]. Table 1 summarizes the baseline characteristics of the included studies, and Table S2 provides a standardized summary of the procedural characteristics of the SNB protocols. Pooled baseline patient characteristics could not be calculated owing to incomplete reporting. Risk-of-bias assessments are presented in Tables S4 and S5. Publication bias was assessed for the pooled rate of subjective improvement in cough severity, as the remaining outcomes were reported in <10 studies. No statistically significant funnel-plot asymmetry was detected for subjective improvement (Egger’s test, *p* = 0.052); however, given the borderline result and the small number of predominantly small studies, small-study effects and publication bias cannot be excluded (Figure S1). The certainty of evidence was judged to be very low for all primary outcomes, including Cough Severity Index improvement, subjective improvement, and adverse events, primarily because of the predominance of observational studies, substantial inconsistency, and imprecision. Detailed GRADE assessments are presented in Table S6.

### 3.1. Changes in Cough Severity Index After SLB

**Figure 2:**
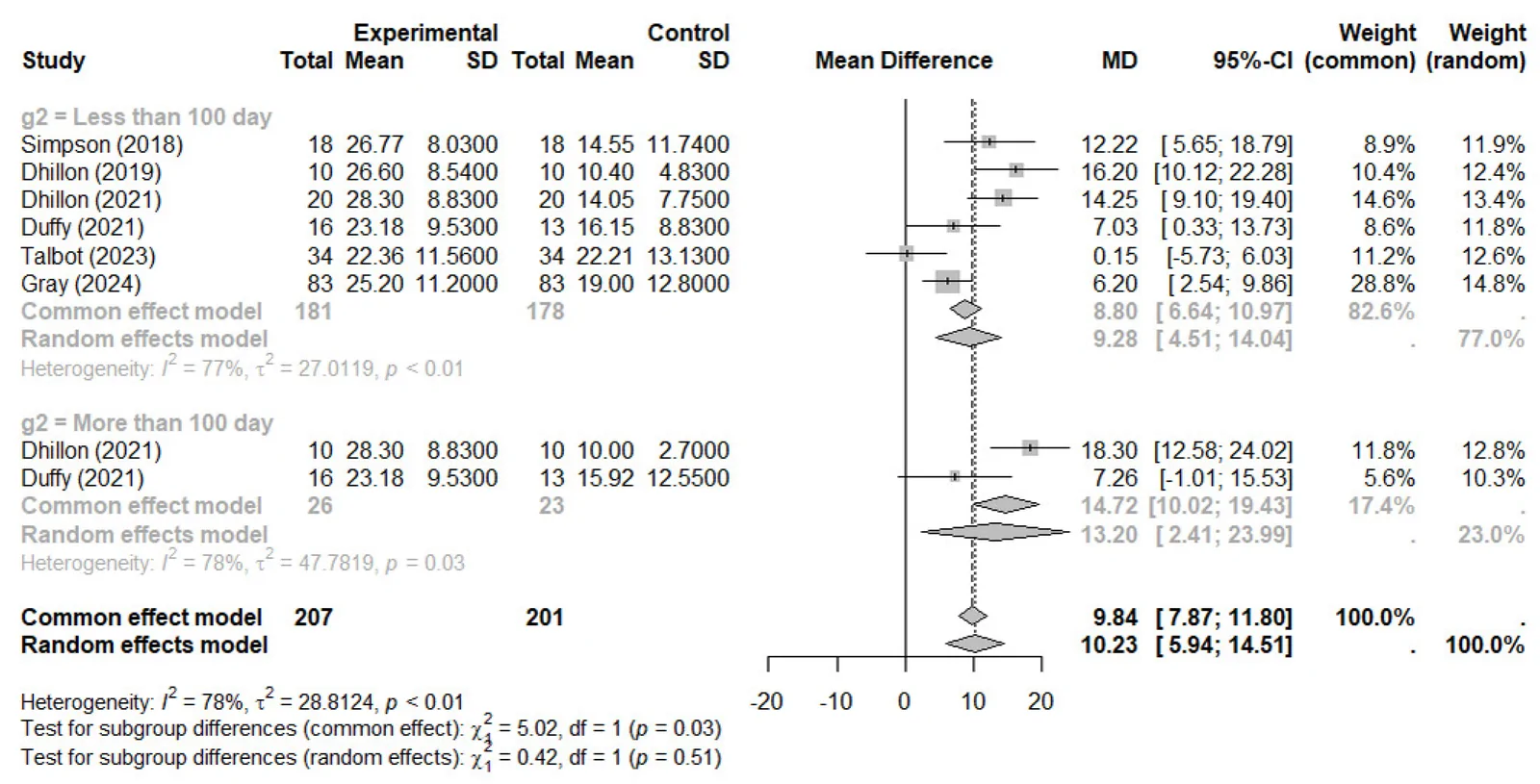
Change in Cough Severity Index (CSI) from pre- to post-treatment with superior laryngeal nerve block (SLB). Values indicate the total number of participants per group [14,15,17,19,20,26].

Using a random-effects model and the change-score approach with an assumed pre–post correlation coefficient (r) of 0.50, SLB was associated with a significant reduction in CSI (MD 10.08; 95% CI 5.79–14.37; I^2^ = 87.5%; Figure 2). Subgroup analysis by follow-up duration showed a similar magnitude of reduction regardless of timing (<100 days: MD 9.48, 95% CI 4.76–14.19; >100 days: MD 11.88, 95% CI −1.00 to 24.76), with no significant subgroup difference (*p* = 0.7313).

In most studies—except those by Quinton (2024) [21], Wehbi (2024) [23], and Diemer (2025) [24]—concurrent bilateral SLB was avoided to minimize the risk of dysphagia or aspiration associated with sensory impairment of the pharynx and larynx. Typically, patients received an initial unilateral block, and those with insufficient response underwent contralateral SLB after an interval of 2 weeks. All studies except Wehbi (2024) [23] reported an average of more than two blocks per patient.

**Figure 3:**
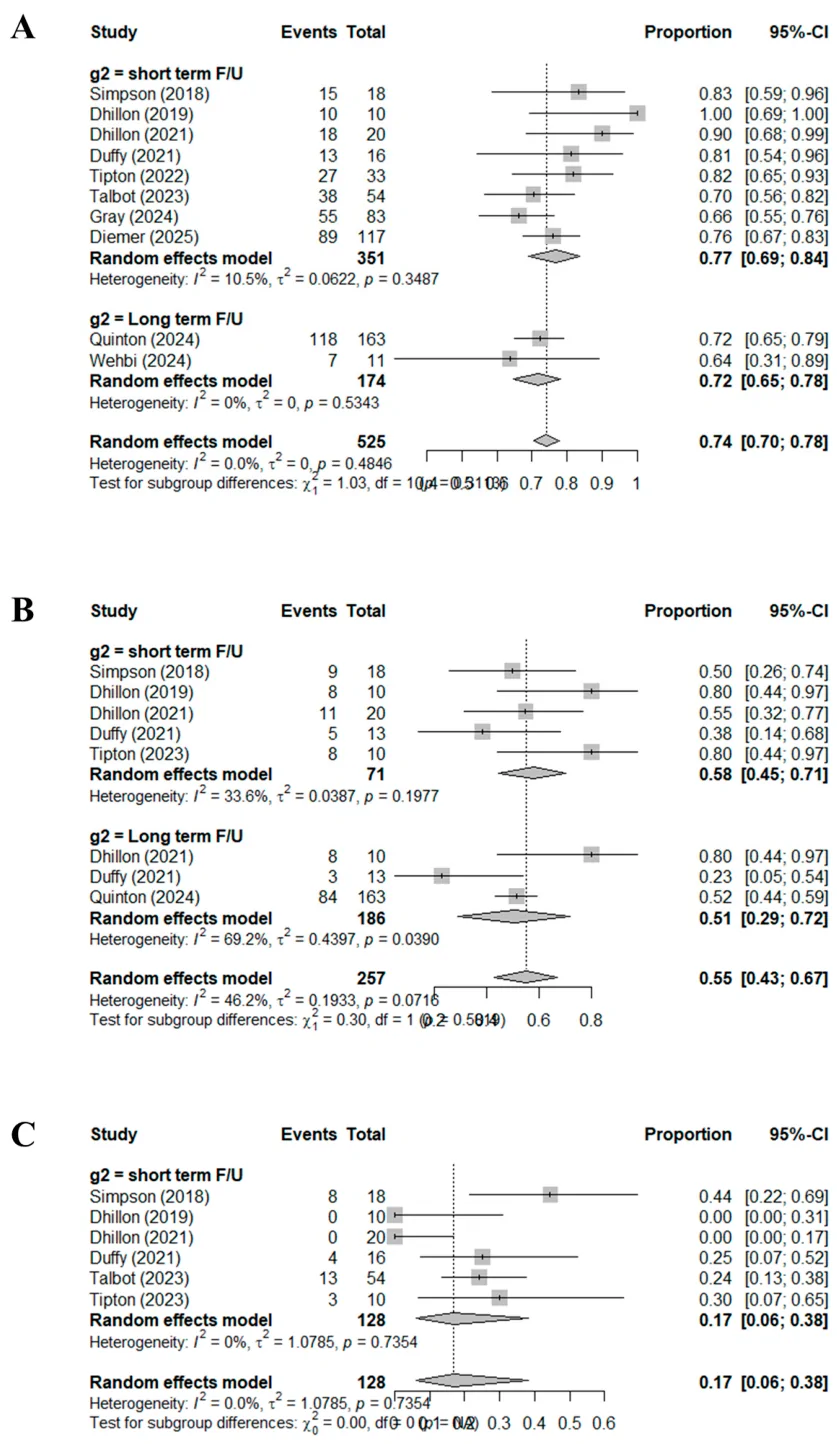
Pooled outcomes. (**A**) Subjective improvement in cough severity, (**B**) ≥50% improvement in cough severity, and (**C**) patient satisfaction or no further need for additional treatment after a single SLB [14,15,17,18,19,20,21,23,24,25,26].

The pooled rate of subjective improvement in cough severity, irrespective of the number of SLB sessions, was 74.29% (95% CI 70.37–77.84; I^2^ = 0.0%; Figure 3A). The pooled rate of ≥50% improvement, based on CSI change or patient self-assessment, was 55.27% (95% CI 42.99–66.94; I^2^ = 46.2%; Figure 3B). Definitions of responder outcomes varied slightly across studies; most reported the ≥50% improvement proportion directly, while two presented responder data graphically or categorically, from which counts were extracted. The proportion of patients reporting satisfaction or no further need for treatment after a single SLB was 16.80% (95% CI 6.19–38.19; I^2^ = 0.0%; Figure 3C).

In the only placebo-controlled randomized trial, saline injection was used as the placebo control. Among 10 patients receiving SLB, eight (80%) reported improvement after ≥1 injection, compared with one of seven patients (14.3%) in the placebo group (*p* < 0.0001). Patients in the treatment group demonstrated significantly lower visual analog scale scores for cough severity (*p* = 0.01) and a mean LCQ score increase from 10.09 to 13.15 (*p* = 0.03), whereas the placebo group exhibited no significant change (8.92–9.54; *p* = 0.44). These findings suggest that SLB may offer greater efficacy than a placebo, although this should be interpreted cautiously given that only one study provided a direct sham-controlled comparison.

### 3.2. Adverse Effects After SLB

**Figure 4:**
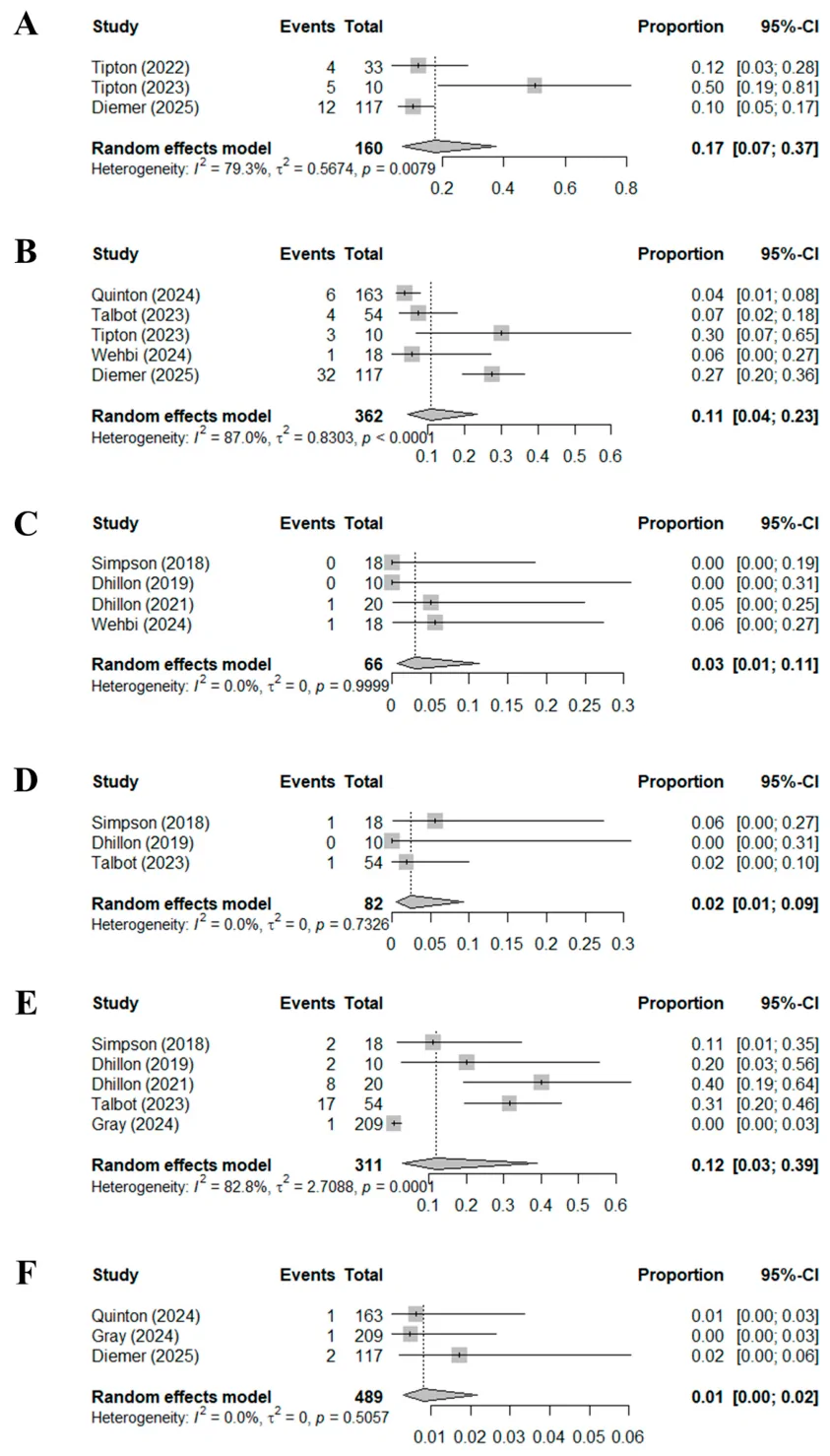
Pooled incidence of adverse effects following superior laryngeal nerve block (SLB): (**A**) globus sensation, (**B**) injection-site pain, (**C**) injection-site swelling, (**D**) mild laryngospasm, (**E**) throat or laryngeal paresthesia, and (**F**) cutaneous purpura [14,17,18,19,20,21,23,24,25,26].

No serious adverse events, aspiration events, procedure-related hospitalizations, or permanent complications were reported across the included studies. Most reported adverse events were mild, transient, and self-limiting, and none resulted in refusal of subsequent injections. The pooled incidence rates were: globus sensation, 17.36% (95% CI 6.86–37.49; I^2^ = 79.3%); injection-site pain, 10.69% (95% CI 4.46–23.45; I^2^ = 87.0%); injection-site swelling, 3.03% (95% CI 0.76–11.32; I^2^ = 0.0%); mild laryngospasm, 2.44% (95% CI 0.61–9.23; I^2^ = 0.0%); throat or laryngeal paresthesia, 11.77% (95% CI 2.72–38.86; I^2^ = 82.8%); and cutaneous purpura, 0.82% (95% CI 0.31–2.16; I^2^ = 0.0%) (Figure 4). A small number of patients developed steroid-related cutaneous purpura or skin fragility following repeated corticosteroid injections; these events resolved after discontinuation or modification of treatment.

### 3.3. Sensitivity Analysis

Leave-one-out sensitivity analysis demonstrated that the pooled effect estimate remained statistically significant after sequential omission of each individual study. The pooled mean difference ranged from 9.78 to 10.69, and all analyses remained highly significant (all *p* < 0.0001). Although the degree of heterogeneity varied slightly (I^2^ 82.7–89.2%), no single study substantially influenced the overall treatment effect, indicating that the findings were robust (Figure S2). Sensitivity analyses using assumed pre–post correlation coefficients of 0.25, 0.50, and 0.75 yielded highly consistent pooled estimates (MD 10.13, 10.08, and 10.02, respectively; Table S7), confirming that the CSI treatment effect was robust to plausible assumptions regarding the within-participant correlation.

## 4. Discussion

Our meta-analysis found that SLB was associated with reductions in cough symptom severity and favorable patient-reported response rates. However, these findings were derived predominantly from uncontrolled observational studies and should not be interpreted as definitive evidence of a causal treatment effect. Because most included studies used a pre–post design without a control group, the observed improvement may have been influenced by placebo effects, spontaneous fluctuation in chronic cough severity, regression to the mean, concomitant therapies, repeated clinical contact, and patient or clinician expectations. These concerns are particularly relevant because the principal outcomes, including the CSI and subjective response rates, were patient-reported and potentially susceptible to expectation bias. Although the pooled reduction in CSI exceeded 10 points, its clinical significance remains uncertain, as a minimal clinically important difference has not yet been established for this instrument. The pooled reduction in CSI was also robust across plausible assumptions regarding the pre–post correlation, supporting the internal consistency of this estimate, though robustness to statistical assumptions does not by itself establish a causal treatment effect. However, interpretation according to follow-up duration requires caution. Although the longer-follow-up subgroup showed a numerically substantial reduction in CSI, its confidence interval was wide and crossed the null because only two study estimates were available and heterogeneity was considerable. The non-significant test for subgroup differences indicates that there was no evidence of a difference between shorter and longer follow-up, but it does not establish equivalent or durable effectiveness over time. To provide additional clinical context, we also evaluated patient-centered outcomes. Approximately 74% of patients reported subjective improvement, and 55% achieved at least a 50% reduction in cough severity, indicating that favorable patient-reported outcomes were observed across multiple measures, in addition to the statistically significant change in CSI; however, these proportions are subject to the same methodological limitations discussed below and should not be equated with confirmed treatment efficacy. However, the pooled proportion of subjective improvement should be interpreted with caution. In most studies, this outcome was assessed using a simple binary patient-reported response (yes/no) rather than a standardized, validated instrument and was therefore susceptible to expectation bias, lack of blinding, interviewer effects, and differences in clinical practice. Accordingly, subjective improvement should be interpreted as a complementary patient-reported outcome alongside validated symptom measures, such as the CSI and, where available, the Leicester Cough Questionnaire and cough visual analog scale, rather than as independent confirmation of treatment efficacy. The only placebo-controlled randomized trial demonstrated greater improvement following superior laryngeal nerve block than saline injection, providing the only controlled evidence supporting a therapeutic effect beyond the observational findings. However, because only one randomized trial was available, the overall certainty of evidence remains very low, and this single trial cannot be considered confirmatory. Although most included studies diagnosed chronic neurogenic cough using a diagnosis-of-exclusion approach after evaluation for common causes of chronic cough, the diagnostic algorithms and previous treatment protocols were not completely standardized across studies. Consequently, residual differences in patient populations and treatment refractoriness may have contributed to the observed clinical heterogeneity. On average, patients required more than two superior laryngeal nerve block procedures to achieve symptom control. Although only a minority experienced satisfactory benefit after a single block, the currently available evidence suggests that superior laryngeal nerve block is generally well tolerated. No aspiration events, procedure-related hospitalizations, or permanent complications were reported in the available studies. However, these findings should be interpreted cautiously because the current evidence is derived predominantly from retrospective observational studies with relatively short follow-up, and the cumulative sample size remains insufficient to reliably detect uncommon or delayed adverse events. Reported adverse events were predominantly mild and transient, including globus sensation (17%), injection-site pain (11%), paresthesia (12%), swelling (3%), mild laryngospasm (2%), and cutaneous purpura (<1%). Furthermore, substantial heterogeneity was observed in the reported incidence of several minor adverse events, particularly globus sensation, injection-site pain, and paresthesia. This variability likely reflects differences in adverse event ascertainment, patient questioning, procedural techniques, and injectate composition rather than true differences in the safety profile.

In many patients, chronic unexplained or treatment-refractory cough is thought to involve sensory neuropathy—commonly referred to as neurogenic cough—proposed to arise, at least in part, from post-viral injury to the internal branch of the superior laryngeal nerve (SLN), which provides supraglottic sensory innervation [17,27]. Although the precise pathophysiology remains incompletely understood, the proposed mechanism is thought to involve heightened cough reflex sensitivity mediated by peripheral and central neural mechanisms. In refractory cases, upregulation of P2X3 receptors on sensory nerve endings has been proposed to contribute to hypersensitivity, whereas C-fiber sprouting may be associated with allodynia and hypertussia. Impaired central inhibitory control may further amplify the reflex, and viral injury may preferentially compromise C-fiber function [28,29]. Even in the absence of a clear viral insult, neural injury may lower the activation threshold of cough receptors. Associated symptoms—including globus sensation, throat clearing, tension dysphonia, and dysphagia—have been suggested to support a neuropathic mechanism [7,30,31]. Management strategies for neurogenic cough include behavioral interventions and pharmacological therapy [7]. Neuromodulators, such as gamma-aminobutyric acid analogs (gabapentin, pregabalin), tricyclic antidepressants (amitriptyline), and opioids, are commonly used [7,9,20]. Among these, gabapentin is considered the standard pharmacological option, although the supporting evidence remains of low quality (Grade 2C, weak recommendation); multimodal speech therapy carries the same recommendation [2].

SLB has been proposed as a candidate intervention for neurogenic chronic cough. Historically, SLB was employed to induce regional airway anesthesia during awake endotracheal intubation [32]; however, it has been reported to provide symptomatic relief in neurogenic cough without the systemic adverse effects associated with oral neuromodulators, such as dry mouth, dizziness, somnolence, sedation, confusion, nausea, blurred vision, headache, and memory impairment [14,17,18,25]. The proposed mechanism is thought to involve a temporary reduction in pathological sensory input from the internal branch of the SLN, thereby raising the threshold for cough reflex activation. However, the exact mechanism of action, the relative contributions of the local anesthetic and corticosteroid components, and the duration of any neuromodulatory effect have not been established and warrant further investigation. Technically, the injection is administered at the point where the nerve traverses the thyrohyoid membrane near the inferolateral hyoid bone. Although no major complications have been reported with SLB, a theoretical risk of severe events—such as blindness or stroke due to embolization of particulate steroids into the arterial circulation—remains. To mitigate this risk, aspiration prior to injection is essential to exclude intravascular placement, and the injectate should be administered slowly.

Overall, the available evidence is consistent with a potential benefit of SLB for refractory chronic cough, although, given the predominance of uncontrolled observational studies, this should be regarded as hypothesis-generating rather than confirmatory. However, reduced efficacy has been reported in patients with comorbid conditions, such as asthma, chronic bronchitis, or chronic obstructive pulmonary disease [22]. This diminished response may reflect concomitant use of multiple medications in these populations or the fact that cough of lower airway origin is predominantly mediated through vagal rather than superior laryngeal nerve pathways [22].

SLB can be administered unilaterally, bilaterally, or at specific trigger points. Although the most commonly used injectate is a 1:1 mixture of triamcinolone and a local anesthetic (1–2% lidocaine or 0.5% bupivacaine, with or without epinephrine), the optimal pharmacologic formulation has not been established. Similarly, the ideal treatment interval remains uncertain. Findings from this meta-analysis suggest that a single injection provides limited benefit, whereas two or more administrations yield more consistent therapeutic effects, though this observation is likewise derived from uncontrolled data. Future comparative and randomized studies are needed to define the most effective dosing regimen and treatment schedule. Moreover, randomized controlled trials are needed to characterize patient-specific response predictors and compare the efficacy of SLB with placebo and other established therapies for chronic cough.

Our study has several limitations. First, all included data were derived from a single region—the United States. Although the U.S. population comprises diverse ethnic groups, including Western, Asian, and African individuals, clinical characteristics of chronic cough and healthcare environments may differ across regions. Second, although overall heterogeneity was low to moderate, clinical heterogeneity among the included studies may have introduced subtle biases. Diagnostic definitions of chronic neurogenic or refractory cough, exclusion criteria for alternative etiologies, injection techniques, medications, injectate volumes, laterality of treatment, treatment intervals, follow-up durations, and the number of superior laryngeal nerve block procedures varied considerably across studies. In addition, the diagnostic evaluation and exclusion criteria for alternative causes of chronic cough were not completely standardized across studies, which may have contributed to residual clinical heterogeneity. Although most studies diagnosed chronic neurogenic cough as a diagnosis of exclusion, the specific diagnostic algorithms and previous treatment protocols were not uniformly reported, limiting direct comparison across studies. Reporting of concomitant therapies during follow-up was also inconsistent. Most studies did not clearly describe whether background treatments, including neuromodulators, speech therapy, or reflux therapy, were continued, discontinued, or modified after SNB. Consequently, the potential contribution of co-interventions to the observed treatment effects cannot be excluded. Therefore, the pooled estimates should be interpreted as reflecting the overall pattern of outcomes associated with superior laryngeal nerve block across diverse clinical protocols rather than the efficacy of any single treatment regimen. As summarized in Table S2, substantial procedural heterogeneity was observed across studies, including differences in corticosteroid formulation, local anesthetic selection, epinephrine use, injectate volume, injection laterality, treatment algorithms, and the number and timing of repeated procedures. These protocol differences preclude identification of an optimal SNB regimen and should be considered when interpreting the pooled results. Despite the observed statistical heterogeneity, both leave-one-out sensitivity analysis and exclusion of the largest study yielded consistent results, indicating that the pooled estimate was robust despite substantial clinical heterogeneity among the included studies, though robustness of the point estimate does not address the underlying risk of bias inherent to uncontrolled designs. At present, the available evidence does not permit identification of an optimal injection protocol, medication, or treatment schedule. Furthermore, meaningful subgroup analyses or meta-regression to further explore potential sources of heterogeneity were not feasible because of the limited number of available studies and substantial overlap in clinical characteristics and treatment protocols. Third, the most important limitation is the low certainty of the primary evidence. Eleven of the 12 included studies were uncontrolled observational studies, and most pooled estimates were based on within-group pre–post comparisons. Such analyses cannot reliably distinguish the treatment effect of SLB from placebo response, natural symptom variability, regression to the mean, concomitant interventions, or expectation-related effects. This limitation is particularly important because chronic cough may fluctuate over time and the principal outcomes were predominantly subjective patient-reported measures. Moreover, several pooled outcomes relied on patient-reported subjective assessments rather than objective cough frequency monitoring. These findings may have been influenced by expectation bias and should be interpreted alongside validated symptom questionnaires rather than as objective measures of treatment efficacy. Another limitation is that objective cough frequency monitoring was not routinely reported in the included studies. Future randomized trials should incorporate objective cough monitoring in addition to validated patient-reported outcome measures. Although the only placebo-controlled randomized trial demonstrated a favorable result for SLB, its small sample size substantially limits the precision and generalizability of that finding. Fourth, long-term outcomes beyond 12 months were not uniformly reported. The available evidence remains insufficient to establish the long-term safety profile of SLB. Rare complications may not have been detected because most included studies were retrospective, involved relatively small sample sizes, and had limited follow-up durations. Finally, cost-effectiveness and health-economic impact analyses were lacking, though such data are critical to guide broader implementation in resource-limited settings.

## 5. Conclusions

Although the available evidence suggests that SLB is associated with clinically meaningful improvement in refractory chronic cough and is generally well tolerated, the certainty of the available evidence is very low because the current evidence base is dominated by uncontrolled observational studies. Published studies have reported predominantly mild, transient adverse events, with no reported aspiration events, procedure-related hospitalizations, or permanent complications. However, larger prospective studies with longer follow-up are required to fully characterize uncommon or delayed adverse events. The available randomized evidence is encouraging but remains limited to a single placebo-controlled trial. Accordingly, adequately powered, well-designed placebo-controlled randomized trials with standardized efficacy and safety outcome assessment are required to confirm the efficacy and safety of SLB.

## Figures and Tables

**Table 1:** Characteristics of studies included in the meta-analysis.

Study (Year)	Study Design	Sample Size	Age (Mean, Range, or Standard Deviation)	Sex (Male/Female)	Nation	Patients Included	Number of Administrations (Mean, Range)	Medication	Follow-Up	Outcomes
Simpson (2018) [14]	Cohort	18	60 (29–86)	3/15	USA	Chronic neurogenic cough	Unilateral: 13 patients bilateral: 5 patients. Mean 2.4 (1–7) block	1:1 mixture of a long-acting particulate corticosteroid and a local anesthetic (2 mL of a 50:50 solution of a long-acting particulate steroid (triamcinolone acetonide 200 mg/5 mL or methylprednisolone 80 mg/1 mL)) and a local anesthetic (1% lidocaine with 1:100,000 epinephrine or 0.5% bupivacaine)	85.4 (7–450) days	Cough Severity Index score, rate of cough improvement, adverse effect
Dhillon (2019) [17]	Cohort	10	54 (34–71)	3/7	USA	Chronic neurogenic cough	Mean 2.3 block	2 mL of a 50:50 solution of triamcinolone acetonide 200 mg/5 mL and 1% lidocaine with 1:100,000 epinephrine	3.4 months	Cough Severity Index score, rate of cough improvement, adverse effect
Dhillon (2021) [26]	Cohort	20	53 (30–83)	3/17	USA	Chronic neurogenic cough	Mean 3 (2–8) block	2 mL of a 50:50 solution of triamcinolone acetonide 200 mg/5 mL and 1% lidocaine with 1:100,000 epinephrine	3.1 months	Cough Severity Index score, rate of cough improvement, adverse effect
Duffy (2021) [15]	Cohort	16	61 (38–88)	3/13	USA	Chronic neurogenic cough	Mean 2.3 (1–17) block	2 mL of a 50:50 solution of a long-acting particulate steroid (triamcinolone acetonide 40 mg/1 mL) and a local anesthetic (0.5% bupivacaine)	Short term (19.5 [8–47] days) and long term (284 [128–430] days)	Cough Severity Index score, rate of cough improvement
Tipton (2022) [18]	Cohort	33	63.9 (41–82)	6/27	USA	Chronic neurogenic cough	Mean 3.9 (1–10) block	1–1.5 mL 50:50 mixture of triamcinolone 40 mg and 1% lidocaine with 1:200,000 epinephrine	6.6 (1–4) weeks	Rate of cough improvement
Quinton (2024) [21]	Cohort	163	NR	NR	USA	Chronic refractory cough	Mean 2.97 (1–22) block (bilateral)	A mixture of triamcinolone acetonide 40 mg/mL with 1% lidocaine or saline	22.5 months	Rate of cough improvement, adverse effect
Talbot (2023) [19]	Cohort	54	59.1 ± 13.9	14/40	USA	Chronic neurogenic cough	Mean 2.13 block	2 mL of a 1:1 solution of triamcinolone (40 mg/mL) and 1% lidocaine with 1:100,000 epinephrine	Median 39 days	Cough Severity Index score, rate of cough improvement, adverse effect
Tipton (2023) [25]	RCT	19	56.3	2/15	USA	Chronic neurogenic cough	NR	Treatment: 1–2 mL of a 1:1 triamcinolone 40 mg: 1% lidocaine with 1:200,000 epinephrine)/placebo: 1–2 mL of saline	NR	Rate of cough improvement, adverse effect
Gray (2024) [20]	Cohort	209	58 ± 13	59/150	USA	Chronic neurogenic cough	NR	2 mL of 1:1 mixture of triamcinolone (40 mg/mL) and 0.5% bupivacaine	2- to 4-week follow-up after single injection	Cough Severity Index score, rate of cough improvement, adverse effect
Shah (2024) [22]	Cohort	44	61 ± 11	10/34	USA	Chronic refractory cough (>8 weeks)	Mean 2 block	Either 0.5 mL of 2% lidocaine + 0.5 mL of dexamethasone sodium phosphate, or 1 mL of dexamethasone sodium phosphate + 1 mL of 0.5% bupivacaine	4 weeks after the final block	Rate of cough improvement, adverse effect
Wehbi (2024) [23]	Cohort	56	64.6 ± 14.8	32/24	USA	Chronic neurogenic cough	Mean 1.2 block	1 mL of a 1:1 dilution of triamcinolone acetate (40 mg/cc) and 1% lidocaine with 1:100,000 epinephrine	7.3 (SD = 10.3) months	Rate of cough improvement, adverse effect
Diemer (2025) [24]	Cohort	117	66.1	28/89	USA	Chronic refractory cough (>8 weeks)	Median 3 block (unilateral or bilateral)	1–2 mL 50:50 mixture of triamcinolone 40 mg and 1% lidocaine with 1:200,000 epinephrine	Median 4 weeks	Rate of cough improvement, adverse effect

RCT; randomized clinical trial, NR; not reported.

## Data Availability

The study extraction sheet, analysis dataset, R statistical code, review protocol, and dated amendment history are publicly available through the Open Science Framework (OSF) repository at https://osf.io/tq7yx/ (accessed on 21 August 2025). The English in this document has been checked by at least two professional editors, both native speakers of English. For a certificate, please see: http://www.textcheck.com/certificate/B1f8lv (accessed on 21 August 2025).

## Supplementary Materials

The following supporting information can be downloaded at: https://www.mdpi.com/article/10.3390/medicina62081583/s1.
