# Supplementary material for: Symptom Control Effectiveness of Superior Laryngeal Nerve Block in Patients with Chronic Cough: A Systematic Review and Meta-Analysis
Source: Medicina (Kaunas). 2026 Aug 18;62(8):1583. doi: 10.3390/medicina62081583 (PMC13515083; doi:10.3390/medicina62081583)
Supplement: Supplementary file 1 [file medicina-62-01583-s001.zip › Table S1.pdf]

Table S1. Search terms and queries.

Database	Search	Search terms / queries
PubMed	#1	"Chronic Cough"[Mesh]
	#2	"Chronic Cough"[TW] OR "Chronic Coughs"[TW] OR "Cough, Chronic"[TW] OR "Persistent Cough"[TW] OR "Cough, Persistent"[TW] OR "Persistent Coughs"[TW] OR "Nagging Cough"[TW] OR "Cough, Nagging"[TW] OR "Nagging Coughs"[TW] OR "neurogenic cough"[TW]
	#3	"Chronic Disease"[Mesh] AND "Cough"[Mesh]
	#4	("Laryngeal Diseases"[Mesh] AND "Hypersensitivity"[Mesh]) OR "laryngeal hypersensitivity"[TW]
	#5	"Laryngeal Diseases"[Mesh] AND "Cough"[Mesh]
	#6 Combine	#1 OR #2 OR #3 OR #4 OR #5
	#7	"Nerve Block"[Mesh]
	#8	"Nerve Block"[TW] OR "Block, Nerve"[TW] OR "Blocks, Nerve"[TW] OR "Nerve Blocks"[TW] OR "Chemical Neurolysis"[TW] OR "Chemical Neurolyses"[TW] OR "Neurolyses, Chemical"[TW] OR "Neurolysis, Chemical"[TW] OR "Chemodenervation"[TW] OR "Chemodenervations"[TW] OR "Nerve Blockade"[TW] OR "Blockade, Nerve"[TW] OR "Blockades, Nerve"[TW] OR "Nerve Blockades"[TW] OR "superior laryngeal nerve blocks"[TW]
	#9	"Laryngeal Nerves"[Mesh]
	#10	"Laryngeal Nerves"[TW] OR "Laryngeal Nerve"[TW] OR "Nerve, Laryngeal"[TW] OR "Nerves, Laryngeal"[TW] OR "Laryngeal Nerve, Superior"[TW] OR "Laryngeal Nerves, Superior"[TW] OR "Nerves, Superior Laryngeal"[TW] OR "Nerve, Superior Laryngeal"[TW] OR "Superior Laryngeal Nerve"[TW] OR "Superior Laryngeal Nerves"[TW]
	#11 Combine	#7 OR #8 OR #9 OR #10
	#12 Combine	#6 AND #11
	#13 Limit	#12 NOT ("Animals"[Mesh] NOT "Humans"[Mesh])

Database	Search	Search terms / queries
EMBASE	#1	"chronic cough"/exp
	#2	"Chronic Cough":ti,ab,kw,de OR "Chronic Coughs":ti,ab,kw,de OR "Cough, Chronic":ti,ab,kw,de OR "Persistent Cough":ti,ab,kw,de OR "Cough, Persistent":ti,ab,kw,de OR "Persistent Coughs":ti,ab,kw,de OR "Nagging Cough":ti,ab,kw,de OR "Cough, Nagging":ti,ab,kw,de OR "Nagging Coughs":ti,ab,kw,de OR "neurogenic cough":ti,ab,kw,de
	#3	"chronic disease"/exp AND "coughing"/exp
	#4	("larynx disorder"/exp AND "hypersensitivity"/exp) OR "laryngeal hypersensitivity":ti,ab,kw,de
	#5	"larynx disorder"/exp AND "coughing"/exp
	#6 Combine	#1 OR #2 OR #3 OR #4 OR #5
	#7	"nerve block"/exp

	#8	"Nerve Block":ti,ab,kw,de OR "Block, Nerve":ti,ab,kw,de OR "Blocks, Nerve":ti,ab,kw,de OR "Nerve Blocks":ti,ab,kw,de OR "Chemical Neurolysis":ti,ab,kw,de OR "Chemical Neurolyses":ti,ab,kw,de OR "Neurolyses, Chemical":ti,ab,kw,de OR "Neurolysis, Chemical":ti,ab,kw,de OR "Chemodenervation":ti,ab,kw,de OR "Chemodenervations":ti,ab,kw,de OR "Nerve Blockade":ti,ab,kw,de OR "Blockade, Nerve":ti,ab,kw,de OR "Blockades, Nerve":ti,ab,kw,de OR "Nerve Blockades":ti,ab,kw,de OR "superior laryngeal nerve blocks":ti,ab,kw,de
	#9	"laryngeal nerve"/exp
	#10	"Laryngeal Nerves":ti,ab,kw,de OR "Laryngeal Nerve":ti,ab,kw,de OR "Nerve, Laryngeal":ti,ab,kw,de OR "Nerves, Laryngeal":ti,ab,kw,de OR "Laryngeal Nerve, Superior":ti,ab,kw,de OR "Laryngeal Nerves, Superior":ti,ab,kw,de OR "Nerves, Superior Laryngeal":ti,ab,kw,de OR "Nerve, Superior Laryngeal":ti,ab,kw,de OR "Superior Laryngeal Nerve":ti,ab,kw,de OR "Superior Laryngeal Nerves":ti,ab,kw,de
	#11 Combine	#7 OR #8 OR #9 OR #10
	#12 Combine	#6 AND #11
	#13 Limit	#12 NOT ('animal'/exp NOT 'human'/exp)

Database	Search	Search terms / queries
Scopus	#1	TITLE-ABS-KEY ("Chronic Cough" OR "Chronic Coughs" OR "Persistent Cough" OR "Persistent Coughs" OR "Nagging Cough" OR "Nagging Coughs" OR "neurogenic cough" OR "laryngeal hypersensitivity")
	#2	TITLE-ABS-KEY ("Nerve Block" OR "Nerve Blocks" OR "Chemical Neurolysis" OR "Chemical Neurolyses" OR "Neurolysis, Chemical" OR "Chemodenervation" OR "Chemodenervations" OR "Nerve Blockade" OR "Nerve Blockades" OR "superior laryngeal nerve blocks" OR "Laryngeal Nerves" OR "Laryngeal Nerve" OR "Superior Laryngeal Nerve" OR "Superior Laryngeal Nerves")
	#3 Combine	#1 AND #2

Database	Search	Search terms / queries
Cochrane Library	#1	[mh "Chronic Cough"]
	#2	"Chronic Cough":ti,ab,kw OR "Chronic Coughs":ti,ab,kw OR "Cough, Chronic":ti,ab,kw OR "Persistent Cough":ti,ab,kw OR "Cough, Persistent":ti,ab,kw OR "Persistent Coughs":ti,ab,kw OR "Nagging Cough":ti,ab,kw OR "Cough, Nagging":ti,ab,kw OR "Nagging Coughs":ti,ab,kw OR "neurogenic cough":ti,ab,kw
	#3	[mh "Chronic Disease"] AND [mh "Cough"]
	#4	([mh "Laryngeal Diseases"] AND [mh "Hypersensitivity"]) OR "laryngeal hypersensitivity":ti,ab,kw
	#5	[mh "Laryngeal Diseases"] AND [mh "Cough"]
	#6 Combine	#1 OR #2 OR #3 OR #4 OR #5

	#7	[mh "Nerve Block"]
	#8	"Nerve Block":ti,ab,kw OR "Block, Nerve":ti,ab,kw OR "Blocks, Nerve":ti,ab,kw OR "Nerve Blocks":ti,ab,kw OR "Chemical Neurolysis":ti,ab,kw OR "Chemical Neurolyses":ti,ab,kw OR "Neurolyses, Chemical":ti,ab,kw OR "Neurolysis, Chemical":ti,ab,kw OR "Chemodenervation":ti,ab,kw OR "Chemodenervations":ti,ab,kw OR "Nerve Blockade":ti,ab,kw OR "Blockade, Nerve":ti,ab,kw OR "Blockades, Nerve":ti,ab,kw OR "Nerve Blockades":ti,ab,kw OR "superior laryngeal nerve blocks":ti,ab,kw
	#9	[mh "Laryngeal Nerves"]
	#10	"Laryngeal Nerves":ti,ab,kw OR "Laryngeal Nerve":ti,ab,kw OR "Nerve, Laryngeal":ti,ab,kw OR "Nerves, Laryngeal":ti,ab,kw OR "Laryngeal Nerve, Superior":ti,ab,kw OR "Laryngeal Nerves, Superior":ti,ab,kw OR "Nerves, Superior Laryngeal":ti,ab,kw OR "Nerve, Superior Laryngeal":ti,ab,kw OR "Superior Laryngeal Nerve":ti,ab,kw OR "Superior Laryngeal Nerves":ti,ab,kw
	#11 Combine	#7 OR #8 OR #9 OR #10
	#12 Combine	#6 AND #11
	#13 Limit	#12 NOT ([mh "Animals"] NOT [mh "Humans"])

Database	Search	Search terms / queries
Web of Science	#1	TS=("Chronic Cough" OR "Chronic Coughs" OR "Persistent Cough" OR "Persistent Coughs" OR "Nagging Cough" OR "Nagging Coughs" OR "neurogenic cough" OR "laryngeal hypersensitivity")
	#2	TS=("Nerve Block" OR "Nerve Blocks" OR "Chemical Neurolysis" OR "Chemical Neurolyses" OR "Neurolysis, Chemical" OR "Chemodenervation" OR "Chemodenervations" OR "Nerve Blockade" OR "Nerve Blockades" OR "superior laryngeal nerve blocks" OR "Laryngeal Nerves" OR "Laryngeal Nerve" OR "Superior Laryngeal Nerve" OR "Superior Laryngeal Nerves")
	#3 Combine	

Database	Search	Search terms / queries
Google Scholar	#1	"Chronic Cough" OR "Chronic Coughs" OR "Persistent Cough" OR "Persistent Coughs" OR "Nagging Cough" OR "Nagging Coughs" OR "neurogenic cough" OR "laryngeal hypersensitivity"
	#2	"Nerve Block" OR "Nerve Blocks" OR "Chemical Neurolysis" OR "Chemical Neurolyses" OR "Neurolysis, Chemical" OR "Chemodenervation" OR "Chemodenervations" OR "Nerve Blockade" OR "Nerve Blockades" OR "superior laryngeal nerve blocks" OR "Laryngeal Nerves" OR "Laryngeal Nerve" OR "Superior Laryngeal Nerve" OR "Superior Laryngeal Nerves"

	#3 Combine	("Chronic Cough" OR "Chronic Coughs" OR "Persistent Cough" OR "Persistent Coughs" OR "Nagging Cough" OR "Nagging Coughs" OR "neurogenic cough" OR "laryngeal hypersensitivity") AND ("Nerve Block" OR "Nerve Blocks" OR "Chemical Neurolysis" OR "Chemical Neurolyses" OR "Neurolysis, Chemical" OR "Chemodenervation" OR "Chemodenervations" OR "Nerve Blockade" OR "Nerve Blockades" OR "superior laryngeal nerve blocks" OR "Laryngeal Nerves" OR "Laryngeal Nerve" OR "Superior Laryngeal Nerve" OR "Superior Laryngeal Nerves")
