# Supplementary material for: Symptom Control Effectiveness of Superior Laryngeal Nerve Block in Patients with Chronic Cough: A Systematic Review and Meta-Analysis
Source: Medicina (Kaunas). 2026 Aug 18;62(8):1583. doi: 10.3390/medicina62081583 (PMC13515083; doi:10.3390/medicina62081583)
Supplement: Supplementary file 1 [file medicina-62-01583-s001.zip › Table S2.pdf]

Table S2. Standardized summary of superior laryngeal nerve block protocols across included studies.

Study	Follow-up	Corticosteroid	LA (Epi)	Volume	Laterality	Injection strategy	Mean SLBs	Interval	Injection site	Notes
Simpson (2018)[a]	Mean 85.4 d (7–450)	TA 40 or MP 80	1% Lido+Epi (1:100k) or 0.5% Bupi[a]	2 mL (1:1)	U→B	TP-directed; contralateral ~2 wk if inadequate; repeat if transient response	2.4 (1–7)	41.5 d	IB-SLN at THM	No simultaneous B blocks
Dhillon (2019)	Mean 3.4 mo (2–6)	TA 40 (200 mg/5 mL)	1% Lido+Epi (1:100k)	2 mL (1:1)	U→B	TP-guided; contralateral 2–3 wk if no response; repeat q2wk if transient	2.3	14–15 d	IB-SLN at THM	≥1 SLP session; no NM during tx; no simultaneous B
Dhillon (2021)	Mean 5.3 mo (overall); orig. cohort 10 mo	TA 40 (200 mg/5 mL)	1% Lido+Epi (1:100k)	2 mL (1:1)	U→B	TP side (else right); ≥3 SLBs q2–3wk; B at each visit until ≥50% improvement	3.0 (2–8)	14.9 d	IB-SLN at THM	≥1 SLP session; no NM; no simultaneous B
Duffy (2021)	ST: mean 19.5 d<br>LT: mean 284 d	TA 40	0.5% Bupi (no Epi)	2 mL (1:1)	U→B	TP side (else posterior THM); contralateral 2wk if no response; repeat only if transient	2 (1–17)	~2 wk	IB-SLN at posterior THM	Prior cough-suppression tx in most; some on stable NM; no simultaneous B
Tipton (2022)	Mean 6.6 wk (1–44)	TA 40	1% Lido+Epi (1:200k)	1–1.5 mL (1:1)	U→B	Ipsilateral to TP/paresis; contralateral if no response; repeat when benefit wanes	3.9 (1–10)	6.6 wk	THM (hyoid, thyroid ala, sup. cornu landmarks)	72.7% on NM; U preferred (↓ globus/aspiration risk)
Quinton (2024)	Mean 22.5 mo	TA 40	1% Lido, no Epi (or saline)	2–3 mL (1–1.5/side)	B (routine)	Series of 3 SLBs q4–6wk; additional injections for responders	2.97 (1–22)	4–6 wk	SLN bundle at THM	1:1 TA+Lido or saline; U reserved for select cases; NPO ~1h post-

										injection; LA>saline for improvement
Talbot (2023)	Median 39 d	TA 40	1% Lido+Epi (1:100k)	2 mL (1:1)	U→contra/ ipsi/occ. B	2nd SLB ~2wk if partial/no response; contralateral if B/poorly localized sx; ipsilateral if persistent lateralizing sx	2.13	~2 wk (median 24–37.5 d)	IB-SLN at THM (thyroid tubercle/ hyoid horn landmarks)	Protocol per Simpson et al.; 96.3% started U; concurrent NM → lower response
Tipton (2023)[b]	2 wk after each injection	TA 40	1% Lido+Epi (1:200k)	1–2 mL (1:1)	U (repeat ipsi/contra by response)	2 U SLBs ~2wk apart per response algorithm	2	~2 wk	Ant. to sup. thyroid cornu, superficial THM	Only placebo-controlled RCT; NM users excluded; aspiration before injection; B intentionally avoided (aspiration risk)
Gray (2024)	2–4 wk after single injection	TA 40	0.5% Bupi (no Epi)	2 mL (1:1)	Single SLB (laterality per clinical judgment)	Single office-based injection; ST assessment only	1	N/A	IB-SLN at THM; paralaryngeal pain: 1mL SLN entry+ 1mL post. cartilage	Largest cohort; ST effect only; aspiration before injection; adjunct to behavioral tx
Shah (2024)	~4 wk after last SLB	DSP 10	2% Lido or 0.5% Bupi (no Epi)	P1: 0.5mL Lido+ 0.5mL DSP; P2: 1mL	U (TP side if identified, else random)	Reassess 2–4wk; repeat by symptom response	Median 2	Mean 1.47 mo	IB-SLN	TA avoided (particulate embolization risk); 1 transient vasovagal episode

				DSP+ 1mL Bupi						
Wehbi (2024)[c]	Mean 6.6 mo	TA[c] 40	1% Lido+Epi (1:100k)	1 mL (0.5+0.5)	B (typical)	Step-up: NM±cough suppression → B SLB if med tx fails; repeat if recurs; BoNT if no SLB response	1.2 (SLN subgroup)	PRN (symptom recurrence)	5mm inferomedial to hyoid lateral aspect	B routine; SLB after NM failure; nonresponders → laryngeal BoNT
Diemer (2025)	Median 4 wk (1–193)	TA 40	1% Lido+Epi (1:200k)	1–2 mL (1:1)	U→B/contra as needed	TP-guided U; B if bilateral TP; repeat/contralateral if no/waning effect	Median 3 (1–32)	PRN (median effect 3.8 wk)	Ant. to sup. thyroid cornu at THM (hyoid/ala/cornu landmarks)	Aspiration before injection; slow injection to avoid IV; improvement in 74.4% of injections; AE in 8.4%; 2 cases cutaneous purpura

Abbreviations: RCS, retrospective case series; RCC, retrospective cohort study; RLFU, retrospective longitudinal follow-up study; MRCC, multi-institutional retrospective cohort study; RCT, prospective randomized placebo-controlled trial; TA, triamcinolone acetonide; MP, methylprednisolone; DSP, dexamethasone sodium phosphate; Lido, lidocaine; Bupi, bupivacaine; Epi, epinephrine; SLN, superior laryngeal nerve; IB-SLN, internal branch of the SLN; THM, thyrohyoid membrane; U, unilateral; B, bilateral; U→B, initial unilateral injection with staged contralateral/bilateral injection if indicated; TP, trigger point; SLB, superior laryngeal nerve block; NM, neuromodulator; SLP, speech-language pathology; BoNT, botulinum toxin; d, day(s); wk, week(s); mo, month(s); N/A, not applicable; PRN, as needed; all 12 studies used a landmark-guided (non-image-guided) injection technique, and no study used ultrasound or fluoroscopic guidance.

a. Simpson (2018): epinephrine present only in the lidocaine formulation, not with bupivacaine.

b. Tipton (2023) is the only placebo-controlled, prospective randomized trial; all other studies are retrospective.

c. Wehbi (2024) used triamcinolone acetate (not acetonide), a different salt formulation of the same corticosteroid class.
