# Supplementary material for: Symptom Control Effectiveness of Superior Laryngeal Nerve Block in Patients with Chronic Cough: A Systematic Review and Meta-Analysis
Source: Medicina (Kaunas). 2026 Aug 18;62(8):1583. doi: 10.3390/medicina62081583 (PMC13515083; doi:10.3390/medicina62081583)
Supplement: Supplementary file 1 [file medicina-62-01583-s001.zip › Table S3.pdf]

Table S3. Diagnostic work-up and prior treatment before superior laryngeal nerve block.

Study	Diagnostic definition	Mean cough duration pre-SLB	Pulm w/u	GERD w/u	Allergy/ UACS w/u	ACE-I review	Prior SLP/ CST	Prior NM	Prior PPI	Prior inhaled/AH	Evidence of refractory cough & key notes
Simpson (2018)	DxE after multidisc. w/u excluding common causes	84.5 mo	✓ (asthma excl.)	✓ (treated pre-dx)	✓ (UACS/atopy treated)	NR	✓ (std pre-SLB)	✓ Gaba/Preg/Ami/Bac; 6 failed, 4 stable-dose	✓ (>2mo ± fundoplication)	✓ (inhaler, AH, nasal steroid, immunotherapy)	Persistent cough despite multidisc. tx + failed NM; 4 pts continued stable-dose NM during SLB (sensitivity analysis: similar results)
Dhillon (2019)	DxE after multidisc. w/u; cough >8wk, neg. objective testing	10 y	✓ (neg. CXR; asthma tx prn)	✓ (empiric PPI ≥4–6wk)	✓ (empiric AH/nasal steroid)	✓ (med-related causes excl.)	✓ (≥1 session, mean 2.4)	✗ excluded (prior/current NM use)	✓ (post-GI eval)	✓ (inhaler, AH, nasal steroid)	Comorbid-cause/NM-use pts excluded; multidisc. w/u for all
Dhillon (2021)	DxE after multidisc. w/u; cough >8wk	11.5 y	✓ (neg. CXR; refractory after med mgmt)	✓ (prior reflux tx)	✓ (evaluated + treated)	✓ (comorbidity causes excl.)	✓ (≥1 session pre-SLB)	✗ excluded (no concurrent NM)	✓ (prior reflux tx)	✓ (prior asthma/allergy tx)	Refractory to conventional mgmt; no concurrent NM during SLB
Duffy (2021)	DxE after neg. w/u; cough >8wk, refractory to std mgmt	96.5 mo	✓ (neg. pulm w/u)	✓ (prior tx failed)	✓ (prior std tx failed)	NR	✓ (12/16 prior CST)	✓ all failed NM; 3 stable Preg/Gaba/Ami during SLB	✓ (failed)	✓ (failed)	All failed med tx before SLB; 13/16 subjective improvement; 3 continued stable-dose NM
Tipton (2022)	DxE after excl. of untreated etiologies;	NR	✓ (CXR + PFT)	✓ (empiric PPI trial or	✓ (allergy testing)	✓ (ACE-I etc. reviewed; untreated	NR	✓ concurrent 72.7% (24/33)	✓ (prior reflux tx/eval)	NR	24/33 concurrent Ami/Gaba (inadequate control → SLB); authors noted

	cough ≥8wk			esophageal w/u)		causes excl.)					possible synergistic effect
Quinton (2024)	Refractory chronic cough after comprehensive w/u (aerodigestive/allergic/neuro excl.); >8wk	NR	✓	✓	✓	NR	✓ 39.9% prior CST	✓ 91.4% (Gaba/Preg/Ami/Nor)	✓ 91.4%	✓ steroids 47.2%, benzonatate 43.0%, codeine 31.3%, tramadol 38.7%	Failed med mgmt before bilateral SLB; multidisc. w/u; largest cohort at publication (n=163)
Talbot (2023)	DxE; cough >8wk; laryngeal hypersensitivity features recorded, not required	70.2 mo	NR (asthma hx recorded, no std w/u)	NR (hx recorded, no std eval/tx req.)	NR (triggers discussed, no std eval)	NR	NR (described as std option; participation NR)	Concurrent 50% (27/54); agents/failures NR	NR	NR	No standardized/required tx failure before SLB (heterogeneous); concurrent NM assoc. with lower response (51.9% vs 88.9%, p=0.003)
Tipton (2023)	DxE + strict objective exclusion criteria; cough ≥8wk	NR	✓ (abnl PFT/CXR ≤6mo = excl.)	✓ (uncontrolled/+ reflux w/u = excl.: esophagram, 24h-pH, manometry, EGD, RSI>13/ RFS>11)	NR (triggers in hx; no std tx req.)	✓ (ACE-I/ARB use = excl.)	✗ excluded (no prior/concurrent CST)	✗ excluded (current NM use)	✓ (required reflux eval pre-enroll)	NR	Strictest diagnostic exclusion criteria among included studies (highest confidence in true neurogenic cough)
Gray (2024)	DxE (neg. w/u: med effect,	NR	✓ (PFT + imaging typical)	✓ (high-dose PPI trial;	✓ (nasal steroid ≥4wk typical)	✓ (med effect part of	✓ 52% (114/209) ≥1 session	NR (adjunct allowed, not quantified)	✓ (high-dose trial typical)	✓ (nasal steroid ≥4wk)	SLB usually adjunct after incomplete

	pulm, GERD, allergic rhinitis, UACS); cough >8wk			many EGD)		w/u; ACE-I not quantified)					response; peri-injection therapy participation did not affect short-term CSI change
Shah (2024)	Presumed laryngeal hypersensitivity (laryngologist dx); no std algorithm	NR	✓ (comorbidity in 54.5% [24/44]; pre-SLB testing NR)	✓ (GERD in 77.3% [34/44]; 91.2% of these on PPI)	✓ (sinonasal disease in 4.5% [2/44]; 64.4% of pulm/UA disease subset on AH/steroid/anti-inflammatory)	NR (ACE-I not specified)	NR	✓ 11.1% on NM at eval; agents NR	✓ 91.2% of GERD pts	✓ 64.4% (of pulm/UA disease subset)	Comorbidities allowed as concomitant (not required treated); no standardized pretreatment trial required; pulm disease → less improvement
Wehbi (2024)	DxE (cough >8wk, refractory; asthma/GERD/med-related causes excl.)	64.4 mo	✓ (failed asthma tx or asthma excl.)	✓ (failed PPI or GERD excl.)	NR (some allergist referral; no std w/u)	NR (ACE-I not separately reported)	✓ (±NM; planned wkly ×4–6wk; mean 2.7 sessions)	✓ step-up Gaba/Ami/Preg ≤6wk; SLB after failure/intolerance	✓ (failed or GERD excl.)	✓ (failed asthma tx or excl.)	Step-up algorithm; SLB = 2nd-line after med/behavioral failure; 7/11 responded to SLB alone (no BoNT)
Diemer (2025)	DxE (untreated etiologies excluded); cough >8wk	7.3 y	✓ (CXR + PFT)	✓ (esophageal eval if indicated)	✓ (allergy testing pre-SLB)	NR	First-line per protocol; completion NR	✓ Ami/Gaba/Bac/Preg considered first-line pre-SLB	✓ (if indicated)	✓ (standard work-up completed; implied)	Standardized w/u + conventional med tx before SLB; n=117, 479 injections; median 3 inj.; AE in 8.4%

Abbreviations: DxE, diagnosis of exclusion; w/u, work-up; UACS, upper airway cough syndrome; PFT, pulmonary function test(ing); CXR, chest X-ray; PPI, proton pump inhibitor; SLP, speech-language pathology; CST, cough suppression therapy; NM, neuromodulator(s); Gaba, gabapentin; Preg, pregabalin; Ami, amitriptyline; Bac, baclofen; Nor, nortriptyline; AH, antihistamine(s); ACE-I, angiotensin-converting enzyme inhibitor; ARB, angiotensin receptor blocker; EGD, esophagogastroduodenoscopy; RSI, Reflux Symptom Index; RFS, Reflux Finding Score; CSI, Cough Severity Index; UA, upper airway; BoNT, botulinum toxin; AE, adverse event(s); NR, not reported; ✓, evaluated/treated/present; ✗, excluded as an eligibility criterion (i.e., not

permitted).

"ACE-I review" indicates whether use of angiotensin-converting enzyme inhibitors (a recognized cause of chronic cough) was reviewed and, where relevant, discontinued prior to enrollment. The "GERD" column encompasses both diagnostic work-up and empiric or confirmed treatment for gastroesophageal reflux disease, as reported by each study.

Concomitant therapy during follow-up: not reported in most studies. Simpson (2018) and Duffy (2021) explicitly reported that a subset of patients continued stable-dose neuromodulators unchanged during SLB follow-up (sensitivity analyses in both studies showed similar treatment effects with these patients included).

Diagnostic algorithms and prior-treatment requirements were not standardized across studies; this clinical heterogeneity is discussed as a limitation in the main text.
