# Supplementary material for: Symptom Control Effectiveness of Superior Laryngeal Nerve Block in Patients with Chronic Cough: A Systematic Review and Meta-Analysis
Source: Medicina (Kaunas). 2026 Aug 18;62(8):1583. doi: 10.3390/medicina62081583 (PMC13515083; doi:10.3390/medicina62081583)
Supplement: Supplementary file 1 [file medicina-62-01583-s001.zip › Table S4.pdf]

Table S4. Methodological quality assessment of non-randomized cohort studies using the Newcastle–Ottawa Scale (NOS).

Study	Selection[a]				Comparability[b]		Outcome[c]			Newcastle-Ottawa Scale score
	1	2	3	4	1	2	6	7	8	
Simpson (2018)	Yes	No	Yes	Yes	Yes	Yes	Yes	Yes	Yes	8
Dhillon (2019)	Yes	Yes	Yes	Yes	No	No	Yes	Yes	Yes	7
Dhillon (2021)	Yes	Yes	Yes	Yes	No	No	Yes	Yes	Yes	7
Duffy (2021)	Yes	Yes	Yes	Yes	No	No	Yes	Yes	Yes	7
Tipton (2022)	Yes	Yes	Yes	Yes	No	No	Yes	Yes	Yes	7
Quinton (2024)	Yes	No	Yes	Yes	Yes	Yes	Yes	Yes	Yes	8
Talbot (2023)	Yes	No	Yes	Yes	No	No	Yes	Yes	Yes	6
Gray (2024)	Yes	Yes	Yes	Yes	No	No	Yes	Yes	Yes	7
Shah (2024)	Yes	Yes	Yes	Yes	No	No	Yes	Yes	Yes	7
Wehbi (2024)	Yes	Yes	Yes	Yes	No	No	Yes	Yes	Yes	7
Diemer (2025)	Yes	Yes	Yes	Yes	No	No	Yes	Yes	Yes	7

The Newcastle–Ottawa Scale (NOS) for cohort studies evaluates three domains: Selection (maximum 4 stars), Comparability (maximum 2 stars), and Outcome (maximum 3 stars), yielding a maximum score of 9 stars. Studies scoring 7–9, 5–6, and <5 stars were considered to be of high, moderate, and low methodological quality, respectively.

a: Selection (4 items): Representativeness of the exposed cohort; Selection of the non-exposed cohort; Ascertainment of exposure; Demonstration that outcome of interest was not present at start.

b: Comparability (maximum 2 stars): Comparability of cohorts on the basis of study design or analysis.

c: Outcome (3 items): Assessment of outcome; Follow-up long enough; and Adequacy of follow-up.
