# Supplementary material for: Symptom Control Effectiveness of Superior Laryngeal Nerve Block in Patients with Chronic Cough: A Systematic Review and Meta-Analysis
Source: Medicina (Kaunas). 2026 Aug 18;62(8):1583. doi: 10.3390/medicina62081583 (PMC13515083; doi:10.3390/medicina62081583)
Supplement: Supplementary file 1 [file medicina-62-01583-s001.zip › Table S5.pdf]

Table S5. Methodological quality assessment of randomized controlled trials.

Study	Random sequence generatio n	Allocation concealment	Blinding of participants and personnel	Blinding of outcome assessment	Incomplete outcome data addressed	Free of selective reporting	Risk of Bias of randomized studies
Tipton (2023)	Yes	Yes	Yes	Unclear	Unclear	Yes	Unclear

Tipton et al. (2023): Outcome-assessment blinding was rated unclear because the study was single-blinded and did not explicitly report independent blinded outcome assessment, although participants remained blinded and the principal outcomes were pa-tient-reported. Incomplete outcome data were rated unclear because 2 of 9 randomized placebo participants withdrew before the first injection and were excluded from analysis, without a reported intention-to-treat analysis or detailed missing-data handling strategy.
