# Supplementary material for: Symptom Control Effectiveness of Superior Laryngeal Nerve Block in Patients with Chronic Cough: A Systematic Review and Meta-Analysis
Source: Medicina (Kaunas). 2026 Aug 18;62(8):1583. doi: 10.3390/medicina62081583 (PMC13515083; doi:10.3390/medicina62081583)
Supplement: Supplementary file 1 [file medicina-62-01583-s001.zip › Table S6.pdf]

Table S6. GRADE Summary of Findings for superior laryngeal nerve block in chronic neurogenic cough.

Outcome	Studies and participants	Effect estimate	Risk of bias	Inconsistency	Indirectness	Imprecision	Publication bias	Certainty
Change in CSI after SLB	8 studies 241 participants	MD 10.08 95% CI 5.79–14.37	Serious[a]	Serious[b]	Not serious[c]	Not serious[d]	Not assessable (<10 studies)[e]	Very low ⊕◯◯◯
Subjective improvement in cough	10 studies 525 participants	74.29% (95% CI 70.37–77.84); I^2=0%	Serious[f]	Not serious[g]	Serious[h]	Not serious[i]	Not detected; residual concern remains[j]	Very low ⊕◯◯◯
Adverse events following SLB	Outcome-specific number of studies varied	Globus 17.36%; injection-site pain 10.69%; swelling 3.03%; laryngospasm 2.44%; paresthesia 11.77%; purpura 0.82%	Serious[k]	Serious[l]	Not serious[m]	Serious[n]	Not assessable (<10 studies)[e]	Very low ⊕◯◯◯

CSI change

[a] Risk of bias—Serious

The evidence was downgraded for risk of bias because most estimates were derived from uncontrolled retrospective cohort studies. The pooled effect represents within-patient change rather than a controlled treatment effect and may therefore be influenced by regression to the mean, placebo response, natural symptom fluctuation, and concomitant treatment.

[b] Inconsistency—Serious

The evidence was downgraded for inconsistency because statistical heterogeneity was substantial, with I^2 exceeding 75%, and important clinical variation existed in patient selection, diagnostic work-up, injection laterality, number of procedures, injectate composition, and follow-up duration.

[c] Indirectness—Not serious

No serious indirectness was identified because the included populations, intervention, and CSI outcome were directly relevant to the review question.

[d] **Imprecision—Not serious**

The pooled confidence interval did not cross the null and remained consistent across plausible pre–post correlation assumptions. However, the cumulative sample size remained limited.

[e] **Publication bias—Not assessable (<10 studies)**

Publication bias could not be formally evaluated because fewer than 10 study estimates contributed to this outcome.

Subjective improvement

[f] **Risk of bias—Serious**

The evidence was downgraded for risk of bias because the outcome was derived predominantly from uncontrolled retrospective studies and was based on unblinded patient reports, which are susceptible to expectation and reporting effects.

[g] **Inconsistency—Not serious**

Statistical inconsistency was not considered serious because the pooled analysis showed $I^2=0\%$.

[h] **Indirectness—Serious**

The evidence was downgraded for indirectness because subjective improvement was generally assessed using a non-standardized binary or chart-derived response rather than a uniform validated outcome measure, and responder definitions varied across studies.

[i] **Imprecision—Not serious**

Imprecision was not considered serious because the pooled estimate had a relatively narrow 95% confidence interval.

[j] **Publication bias—Not detected, but cannot be excluded**

Egger's test was not statistically significant ($p = 0.052$), but the borderline result, limited number of studies, and predominance of small single-center cohorts mean that small-study effects and selective publication cannot be excluded.

Adverse events

[k] **Risk of bias—Serious**

The evidence was downgraded for risk of bias because adverse events were predominantly collected retrospectively, and methods of active surveillance, questioning, and documentation were not standardized.

[l] **Inconsistency—Serious**

The evidence was downgraded for inconsistency because substantial heterogeneity was observed for several adverse events, including globus sensation, injection-site pain, and paresthesia.

[m] **Indirectness—Not serious**

The reported adverse events were directly relevant to the safety of SLNB in the target population.

[n] **Imprecision—Serious**

The evidence was downgraded for imprecision because individual adverse events were uncommon, confidence intervals were wide for several outcomes, and event rates were low; cumulative sample size and follow-up were inadequate to reliably detect rare or delayed complications.

Abbreviations: CSI, Cough Severity Index; CI, confidence interval; MD, mean difference; SLNB, superior laryngeal nerve block.
