# Supplementary material for: Symptom Control Effectiveness of Superior Laryngeal Nerve Block in Patients with Chronic Cough: A Systematic Review and Meta-Analysis
Source: Medicina (Kaunas). 2026 Aug 18;62(8):1583. doi: 10.3390/medicina62081583 (PMC13515083; doi:10.3390/medicina62081583)
Supplement: Supplementary file 1 [file medicina-62-01583-s001.zip › Table S7.pdf]

Table S7. Sensitivity analyses using different assumed pre–post correlation coefficients for estimation of the standard deviation of the change score.

Assumed correlation coefficient (*r*)	Pooled MD	95% CI	*p* value	I^2
0.25	10.13	5.82–14.44	<0.0001	82.60%
0.50 (primary analysis)	10.08	5.79–14.37	<0.0001	87.50%
0.75	10.02	5.76–14.29	<0.0001	92.80%

The SD of the change score was estimated using assumed within-participant pre–post correlation coefficients (*r*) of 0.25, 0.50, and 0.75.

Abbreviations: CI, confidence interval; CSI, Cough Severity Index; I^2, inconsistency statistic; MD, mean difference.
